# 4163 Aging and Smoking Exacerbates Post-Stroke Complement Driven Neuroinflammation

**DOI:** 10.1017/cts.2020.50

**Published:** 2020-07-29

**Authors:** Christine Couch

**Affiliations:** 1 Medical University of South Carolina

## Abstract

OBJECTIVES/GOALS: Following stroke, complement-dependent neuroinflammation exacerbates secondary injury and worsens acute and chronic outcomes. We have shown that an injury site-targeted complement inhibitor (B4Crry), that targets specifically to the ischemic brain, inhibits complement activation leading to improved outcomes. Stroke comorbidities have been shown to promote a pro-inflammatory environment in the brain and systemically, and to exacerbate inflammatory responses after injury. We investigated the impact of age and smoking on acute outcomes after stroke and assessed whether increased complement activation contributes to the worsening outcomes with these stroke comorbidities. METHODS/STUDY POPULATION: Mouse brain endothelial cells (bEnd3) were exposed to hypoxia followed by exposure to serum that was derived from either cigarette smoke (CS)-exposed mice or naïve mice, and IgM and C3d deposition assessed. Adult (12 weeks) and aged (1 year) mice were subjected to 1h transient middle cerebral artery occlusion. Animals were exposed to CS for 3-6 months (5hr/day, 5days/week) by burning 3R4F cigarettes using a smoking machine. Animals were treated with B4Crry or vehicle intravenously 2h post-MCAO. Survival analysis and neurological deficit scores were performed up to 7 days. Brains were extracted for histological and molecular analyses. RESULTS/ANTICIPATED RESULTS: Following hypoxia, bEnd3 cells exposed to serum from CS-exposed mice had higher C3d and IgM deposition compared to naïve serum. Older and CS-exposed mice had significantly worse neurological deficits and mortality compared to younger adults post-MCAO. B4Crry reduced mortality and motor deficits in young, old and old+CS mice with a higher effect size in comorbid animals. Age and/or CS exposure resulted in larger infarct volumes, and increased levels of C3d deposition and microglial activation compared to young adults, but aged/CS animals treated with B4Crry fared comparable to young adults. DISCUSSION/SIGNIFICANCE OF IMPACT: The pro-inflammatory effects of aging and smoking contribute to worse stroke outcomes, and these effects can be successfully mitigated by injury site-targeted complement inhibition.

